# The EPIC Kids Study: a randomized family-focused YMCA-based intervention to prevent type 2 diabetes in at-risk youth

**DOI:** 10.1186/s12889-015-2595-3

**Published:** 2015-12-18

**Authors:** Melanie D. Hingle, Tami Turner, Randa Kutob, Nirav Merchant, Denise J. Roe, Craig Stump, Scott B. Going

**Affiliations:** Department of Nutritional Sciences, The University of Arizona, 1177 E 4th St, Shantz Bldg, Room 328, Tucson, AZ 85721 USA; Department of Family & Community Medicine, The University of Arizona, Faculty Office Building #220, Tucson, AZ USA; Arizona Research Laboratories, The University of Arizona, TW Keating Bioresearch Bldg. #240, Tucson, AZ USA; Mel & Enid Zuckerman College of Public Health, The University of Arizona, Leon Levy Cancer Center #222, Tucson, AZ USA; Department of Medicine, The University of Arizona, AHSC #05099, Tucson, AZ USA; Southern Arizona VA Health Care System, Tucson, AZ USA

**Keywords:** Pediatric obesity, Diabetes mellitus, Intervention studies, mHealth

## Abstract

**Background:**

It is well established that behavioral lifestyle interventions resulting in modest weight reduction in adults can prevent or delay type 2 diabetes mellitus; however in children, successful weight management interventions are rarely found outside of controlled clinical settings. The lack of effective community-based programs is a barrier to reducing obesity prevalence and diabetes risk in children. The objective of our study is to develop and test a group-randomized family-centered community-based type 2 diabetes prevention intervention targeting at-risk children, 9- to 12-years-old.

**Methods/Design:**

Using participatory methods, the adult-focused YMCA Diabetes Prevention Program was adapted for families, creating a novel lifestyle behavior change program focused on healthy eating, physical activity, and a supportive home environment. The program will be tested in sixty 9- to 12-year-old children at risk of diabetes and sixty parents over 12 consecutive weeks with two intervention formats randomized by location: a face-to-face instructor-led program, or a hybrid program with alternating face-to-face and mobile technology-delivered content. Anthropometric, behavioral, psychosocial and physiological outcomes will be assessed at baseline, post-intervention (12 weeks), and follow-up (24 weeks). Secondary outcomes are participant acceptability, feasibility, and adherence. The RE-AIM framework (reach, efficacy, adoption, implementation, and maintenance) will guide intervention implementation and evaluation. Changes at 12 weeks will be assessed using a paired t-test combining both delivery formats. Exploratory models using linear regression analysis will estimate the magnitude of the difference between the face-to-face and hybrid format. The sample size of 60 children, informed by a previous YMCA intervention in which −4.3 % change in overweight (SE = 1.1) was observed over 6 months, will give us 80 % power to detect an effect size of this magnitude, assuming a one-sided test at alpha = 0.05.

**Discussion:**

The proposed study capitalizes on a partnership with the YMCA, a popular and widespread community organization, and uses mobile technologies to extend program reach while potentially reducing burden associated with weekly attendance. The long-term goal is to create a scalable, replicable, and sustainable pediatric “diabesity” prevention program that overcomes existing barriers to the translation of efficacious interventions into effective community programs.

**Trial registration:**

ClinicalTrials.gov, NCT02421198 on April 15, 2015

## Background

The statistics are startling: nearly one-third of U.S. children and adolescents are overweight and one in five are obese [1]. Given the strong association between obesity in youth and the risk for chronic disease, and persistence of obesity into adulthood [2, 3], weight control and obesity prevention are critical to type 2 diabetes mellitus (T2D) and cardiovascular disease prevention. In children, short-term weight loss/weight management success has been achieved in controlled clinical and research settings [4, 5]; however, long-term success is rare, adherence is low, and studies outside of these settings often lack comparison groups and clinical outcomes other than body weight and body mass index (BMI) [6]. The paucity of effective obesity prevention programs adapted for community settings represents a significant barrier to reducing obesity prevalence and T2D risk in children.

In adults, behavioral interventions have proved more efficacious than pharmacological interventions for T2D prevention [7] with established evidence for long-term risk reduction [8, 9]. In youth, novel strategies for increasing engagement and supporting parental involvement are needed to achieve similar effects. Studies suggest that effective youth interventions are family-centered [10, 11], target both diet and physical activity [5, 12], and include activities that promote adoption of healthy behaviors by parents who are often also at high risk for T2D [13]. Parents shape their child’s food and physical activity environments, making them central agents for prevention and treatment of weight-related problems. In this role, parents require a combination of information, skills, resources, and opportunities to support their child in healthy behavior change [14, 15].

The proposed program - EPIC Kids (Encourage - Practice - Inspire - Change) will provide critical infrastructure to support youth and families in making lifestyle changes by adapting the successful adult YMCA Diabetes Prevention Program (YDPP) [7, 16] for at-risk (≥85 BMI percentile) children, ages 9- to 12-years-old, and their families. Our goal is to promote youth and family adoption of behaviors associated with a healthy weight trajectory to prevent excess weight gain while supporting normal growth and development in children. Target lifestyle behaviors in support of this goal are to make physical activity an integral and routine part of life, eat a healthy diet (both quality and quantity) and create food and physical activity environments to insure healthy options and behaviors are the routine, easy choice.

The EPIC Kids Study has the following objectives:

1. Adapt the efficacious Diabetes Prevention Program (DPP) in adults – the adult YMCA DPP – for delivery to children, ages 9-12-years-old, and their families
2. Test the impact of the program on child anthropometric, behavioral, physiological, and psychosocial outcomes
3. Assess feasibility, participant acceptance (child and parent), and retention rates of the program using two delivery formats randomized by YMCA location: a 12-week face-to-face program and a 12-week hybrid face-to-face and mobile device-based program

Herein we describe the design and evaluation of the EPIC Kids Study, a family-focused, YMCA-based diabetes prevention program specifically designed for at-risk children and their families.

## Methods/Design

### Study design

**Fig. 1:**
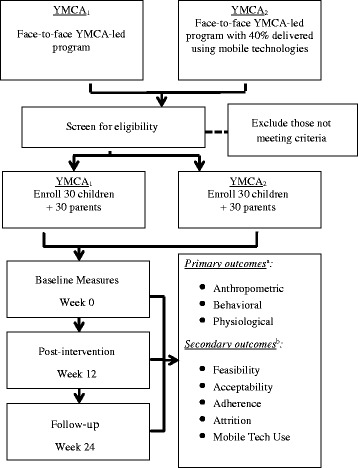
EPIC Kids Study design, recruitment, procedures

The impact of the EPIC Kids intervention will be evaluated using a group-randomized, non-inferiority trial design with three data collection points: baseline, post-intervention (immediately post program completion at 12 weeks), and follow-up (24 weeks) (Fig. 1). The program will be tested with sixty, 9- to 12 year-old children and their parent(s) or primary caregiver(s) at two YMCA locations in Tucson, Arizona where families lack services and resources needed for T2D prevention. Randomization to delivery formats will occur by YMCA location to minimize between-group contacts. One site will conduct a face-to-face weekly YMCA lifestyle coach-led program, while the second site will host a program equivalent in duration, frequency, and content, also lead by YMCA coaches, but with 5 weeks (40 %) of content digitally delivered using study-provided mobile devices (Kindle Fire HDX7, Amazon). Thirty families will be recruited to participate in each intervention format; each family will be assigned to an intervention group led by two trained YMCA lifestyle coaches and consisting of up to 9 other families. Weekly sessions will occur over 1.5 h for 12 weeks.

Baseline, post-intervention (12 weeks), and follow-up (24 weeks) measures will assess changes in child anthropometric, behavioral, psychosocial, and physiological outcomes. We will also determine whether the two delivery formats are acceptable and feasible, and if the use of technology reduces participant burden and improves adherence for the ‘hybrid’ condition. The study protocol was approved by the University of Arizona Institutional Review Board, and is registered with ClinicalTrials.gov (NCT02421198).

### Participants

Eligible participants are 9 to 12-years-old at study enrollment, have a BMI at or greater than the 85th percentile for age and sex, and have one or more of the following T2D risk factors: ethnic minority, first or second degree relative with T2D, conditions associated with insulin resistance or metabolic syndrome (including acanthosis nigricans, hypertension, dyslipidemia, polycystic ovarian disease, small for gestational age birth weight, maternal history of gestational diabetes [17]). Eligible children must also have a primary caregiver willing to participate in intervention sessions and activities (note: primary caregiver is an adult who most frequently prepares/obtains food, regulates media use, and provides physical activity opportunities for the child), be willing to use a study-provided mobile device throughout intervention, and speak and read English. Exclusion criteria are previously diagnosed Type 1 or Type 2 diabetes mellitus (this is not an exclusion criteria for the caregiver), psychiatric disturbances or mental illness, limitations preventing physical activity, or using medications known to cause weight loss or gain or affect appetite.

Participants will be recruited through pediatric and family medicine practices, the Southern Arizona YMCA membership, and the Tucson community (e.g., libraries, community centers, public health department, and local health fairs) using electronic announcements, flyers, posters, and word-of-mouth between May and August 2015, and between October 2015 and January 2016. Respondents will be invited to attend study information sessions held at participating YMCA locations where eligibility will be confirmed. Interested and eligible respondents will complete the informed consent process following University of Arizona Institutional Review Board-approved materials and methods and assigned a study location based on geographical proximity. Written consent will be obtained from parents; verbal assent will be obtained from youth and documented in writing by study staff. Families who are not yet YMCA members will be provided access to YMCA facilities free of charge for the duration of the study, with an opportunity to continue at a reduced rate after the study has concluded.

### Sample size and power

The primary outcome is change in percent overweight. Our estimated sample size was informed by the results of Foster et al. [18] who observed a mean decrease of 4.3 % in percentage overweight (SE = 1.1) in a YMCA-based pediatric obesity intervention over 6 months, and is based on the number of participants needed to detect an effect size of this magnitude with 80 % power assuming a one-sided test at alpha = 0.05. Given a final sample of 48 children and allowing for up to 20 % attrition, 60 participants will be recruited.

### Research setting

The YMCA of Southern Arizona (YMCA-SAZ) will play a significant role in the intervention. As a certified training center for the Y-USA Diabetes Prevention Program (YDPP), YMCA-SAZ administrators and staff have experience delivering the successful adult-focused YMCA Diabetes Prevention Program [8, 16]. Two sites will serve as study locations, at which intervention and assessment activities will be conducted. Both facilities are easily accessible by public transit. Membership demographics indicate substantial minority and underserved membership (~67 % Hispanic, 71 % eligible for free and reduced lunch). Both locations report high family memberships (in the hundreds). Standard amenities at all YMCA locations include full service childcare available to all members free of charge; community rooms and free Wi-Fi throughout; sports fields; cardio wellness centers with state-of-the-art strength and fitness equipment; group exercise facilities; a complete aquatics center; locker rooms; and a space dedicated to youth activities. In addition, both YMCA locations provide sports, recreation, and fitness activities tailored to families. YMCA family memberships will be provided free of charge to all participants for the duration of the study.

### Intervention development

The YMCA’s (adult-focused) Diabetes Prevention Program (YDPP) served as a guide for the development of the EPIC Kids program. The YDPP consists of a series of weekly lessons delivered by trained paraprofessionals at the YMCA over 4 months. Program goals are weight loss through healthy eating and increased physical activity; all activities provide opportunities to learn and practice lifestyle behaviors (e.g., identifying calorie-dense foods; beginning an exercise program). EPIC Kids was designed to retain the relevant, evidence-based features and structural elements of the successful adult-focused program, with two key adaptations related to content and delivery - content specific to youth and families and delivery of content using mobile devices - both designed to address and overcome potential barriers to implementation unique to youth and families.

#### Adaptation 1: Inclusion of content specific to youth and families

Congruent with the 2012 IOM Report [19] and guidelines set forth by an Expert Committee [20], target lifestyle behaviors in support of childhood obesity prevention are 1) make physical activity an integral and routine part of life; 2) eat a healthy diet (both quality and quantity); and 3) create food and physical activity environments to insure healthy options and behaviors are the routine, easy choice. Relevant content and activities drawn from the intervention literature [15, 21] were also integrated, including behavior change techniques associated with successful lifestyle behavior change (e.g., self-monitoring, reinforcement, goal-setting, coping strategies [22]). Curriculum was further enhanced with fun, interactive, and active content designed to provide children and their parents with opportunities to learn and to practice healthy lifestyle behaviors as a family. Parents will receive further support during the program with a series of “parent-only” discussions led by lifestyle coaches which will focus on promoting the use of proactive parenting practices such as role modeling healthy eating and activity, increasing availability/accessibility of healthy foods in the home, and offering frequent opportunities for children to be active [23].

#### Adaptation 2: Delivery of content using mobile devices

Anticipating a ‘hybrid’ intervention format with an alternating face-to-face/digital delivery, five out of twelve EPIC Kids sessions (40 %) were adapted for delivery through the study-provided tablet (Kindle Fire HDX7, Amazon). Best practices for mobile content design and delivery informed these adaptations, with a focus on the persuasive design elements associated with mobile health behavior change (e.g., engaging interface, relevant content, customized prompts for goal-setting, self-monitoring, and social support) [24] (Table 1).

**Table 1:** Program goals, target behaviors, and behavior change techniques

Program goals	Target behaviors	Behavior change techniques
Make physical activity an integral and routine part of life	Encourage moderate-to-vigorous physical activity	Goal setting
	Manage screen time	Self-monitoring
Eat a healthy diet (both quality and quantity)	Promote nutrient dense foods with an emphasis on vegetables, legumes, and whole grains	Role modeling by others (especially parents and siblings)
	Limit energy-dense foods (especially high sugar, high fat snacks)	Social support
	Limit sugar-sweetened beverages	Problem solving
	Encourage adequate sleep	Feedback
Create food and activity environments to insure healthy options and behaviors are the routine, easy choice	Practice proactive food, physical activity, and media parenting	Positive self-talk
	Structure the home environment to support healthy choices	Mindfulness
	Plan ahead for meals (home and restaurants)	
	Make time for family meals, activities, and media	

### Formative research

Participatory methods were used to refine intervention content and delivery methods. Formative research partners included an external advisory board comprised of experts in diet, physical activity, youth development, diabetes, mobile technologies, and pediatric medicine; youth in the target age range (9- to 12-years-old); administrators and program staff at the YMCA of Southern Arizona and Y of the USA, and staff from The University of Arizona Cooperative Extension’s Garden Kitchen, a seed-to-table nutrition education program serving the City of South Tucson.

In September and October 2014, in-depth interviews were conducted with two YMCA administrators and four staff to explore factors influencing local adoption and delivery of EPIC Kids. Discussion topics included branding and licensing issues, and ongoing costs and resources associated with training, implementation, and evaluation. Interviews also explored resources needed to conduct the future large-scale study. Two researchers trained in qualitative procedures conducted the interviews, which were audio-recorded, transcribed, and coded to identify salient issues.

In October 2014 and March 2015, advisory board members reviewed intervention materials and assessed whether proposed topics and activities aligned with program goals, rated the potential of each topic and activity to influence obesogenic behaviors, and insured the proposed activities fostered skill acquisition and provided opportunities for further practice. Following advisory board review, content was revised and further refined by the research team during a series of user tests conducted February to April 2015 with five children aged 9- to 12-years-old recruited from the YMCA. Participants met with the research team during six, 1.5-hour sessions held every other week for twelve weeks. At each session, participants were asked to complete a series of scripted activities mimicking study engagement (e.g., goal-setting, food preparation and sampling, physical activities) and verbalize their reactions (i.e., “think aloud”) as they completed the activities. Participants also viewed sample content from weekly sessions and rated its acceptability, their comprehension, and enjoyment. Following recommended guidelines for qualitative data collection [25], session notes were summarized and coded by the research team to identify salient issues related to content and delivery. Findings from user tests were presented to study investigators and advisory board members, and following a second period of review by these stakeholders, final adjustments to the intervention were completed.

### Intervention structure and content

Each EPIC Kids session is 1.5 h in length; sessions are delivered weekly over 12 consecutive weeks. In addition to incorporating structural elements that promote fun, activity, and interactivity (e.g., hands on experiential learning, kid-led activities) among children, family members and coaches; content is focused on impacting modifiable diabetes risk factors including the home environment, parenting practices, diet quality, physical activity, screen media use, and sleep. Sessions are structured to foster skill building and provide repeated opportunities to practice healthy lifestyle behaviors. Each week has a similar format, beginning with family physical activity, and concluding with goal setting (Table 2); all activities incorporate evidence-based behavior change techniques and behavioral targets.

**Table 2:** EPIC Kids intervention – weekly session format

Activity	Description	Rationale for activity
Drop-in physical activity	A “join-as-you-arrive” physical activity provides a preview of the PA activity of the week	Get kids and families moving instead of sitting upon arrival
Reflection	Groups families together to discuss previous week’s goal-setting to share challenges and successes; self-monitoring is discussed and incentivized	Foster between-family interactions, source of new ideas and inspiration for others, ‘accountability’
Food for thought	Live food demo and tasting opportunity focused on one of the three goal food groups: vegetables, legumes, whole grains	Prepare and taste healthy (and delicious) food
Family physical activity	These are fun physical activities the whole family can enjoy; minimal equipment required	Demonstrate activity can be fun, especially when the entire family gets involved
OR		
Kid physical activity and parenting discussion	Active and kids choices, and FUN; parents focus on a parenting energy balance topic during this moderated discussion	Demonstrate activity can be fun; Parents learn proactive parenting around media, food, and physical activity
Energy balance activity	Hands-on activities that provide families with foundational knowledge and opportunities to practice healthy lifestyle behaviors	Provide opportunities to skill-build and practice as a family
Goal-setting	Integral to behavior change, families work together to set goals around energy balance behaviors	Promote behavior change

### Content for hybrid program

Mobile-enabled content represents an enhancement compared to the traditional face-to-face intervention. Since this innovation is untested in our target population, we used previously established protocols [26] to insure appropriate tailoring for our intended audience – children and families. Our delivery approach is informed by the multimedia learning literature [27], which emphasizes individual differences in rates of learning and behavior change, and the importance of customized program delivery to fit participants’ preferences and abilities.

**Fig. 2:**
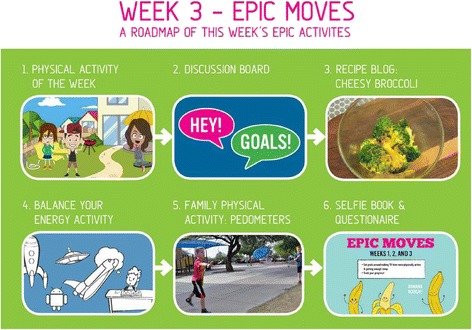
EPIC Kids mobile screen shot – home page for week 3 activities

**Fig. 3:**
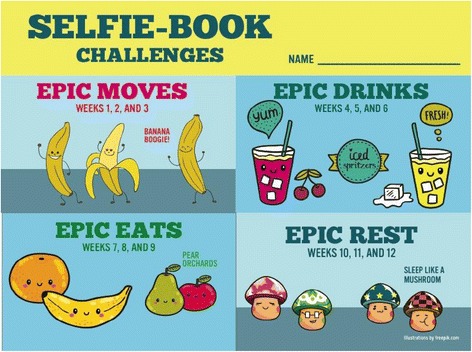
EPIC Kids mobile screen shot – goal-setting and self-monitoring tool

With input from our Advisory Board and instructional media design specialists, the team prepared intervention content for mobile delivery during October 2014 to May 2015. The infrastructure supporting delivery of mobile-enabled content was established *a priori* [28], thus, development activities focused on customization of the interface, and conversion of activities and interactive elements of EPIC Kids to a mobile-friendly format (see Figs. 2 and 3).

### YMCA lifestyle coach training

Twelve YMCA Lifestyle Coaches – paraprofessionals hired by the YMCA to deliver the adult-focused YDPP – will be trained to deliver the EPIC Kids intervention to participating families. Selection of coaches will occur based on a positive recommendation by their YMCA supervisor, their own interest, and prior experience leading group education sessions. In preparation, coaches will complete 16 h of training led by university-based researchers and YMCA staff in July/August and November 2015. During these training sessions, coaches will have the opportunity to learn and practice all aspects of the EPIC Kids curriculum and master competencies necessary to encourage children and parents in lifestyle behavior change including fundamentals of healthy eating and exercise, proactive parenting practices, communication skills (including group facilitation), and organizational skills/time management [29]. Trainings will take place over several sessions; each session will provide a combination of classroom-style didactic lessons and mock group sessions where coaches lead activities while trainers and trainees act as participants. This type and amount of training has been shown effective in translating previous T2D prevention programs to community settings [16, 30].

### Intervention delivery

The intervention will be conducted over 12 weeks at two YMCA locations. Each location will engage up to thirty children and their parents. The program duration is informed by the length of the YMCA adult-focused Diabetes Prevention Program, as well as studies in adults [31, 32] and children [33] that suggest success in weight loss and behavior changes during the first 8 to 12 weeks of a program predict overall success. Each Y location will host up to three concurrent intervention groups (each comprised of no more than 10 families) led by two YMCA Lifestyle Coaches. Program activities will occur at times convenient to parents (typically, early evening or weekend). All activities will promote active learning (i.e., hands-on activities requiring movement, interaction) and provide opportunities to build and practice skills related to healthy eating and physical activity as a family. For participants in the hybrid intervention, content will consist of equivalent topics and activities as the face-to-face sessions, made accessible through the study website (Moodle Pty Ltd, Perth, Australia) and a study-provided mobile device (Kindle Fire HDX7, Amazon).

## Procedures

### Randomization

Two YMCA sites, [A] and [B], were randomly assigned to face-to-face versus hybrid intervention formats using a random number table. Based on alphabetical order and an even number selected, [A] was assigned to receive the face-to-face format and [B] was assigned the hybrid format.

### Data collection

Anthropometric, behavioral, psychosocial and physiological outcomes will be evaluated at baseline, immediately following the 12-week intervention, and at follow-up (24 weeks). At each of these time points, weight will be measured using an electronic calibrated scale (Seca 876, Chino, CA) and rounded to the nearest 0.1 kg with participants wearing light clothing and no shoes. Height will be measured by a portable stadiometer (ShorrBoard, Olney, MD) and rounded19 to the nearest 0.1 cm. Waist circumference, linked to metabolic syndrome in children [34], will be measured at the umbilicus using standard protocols [35]. All anthropometric measures will be taken in duplicate and averaged. Lacking a gold standard for measuring change in weight status in children, we will use the recommended change in percentage overweight [36] calculated as percentage over the median BMI for age and gender. Child dietary intake will be assessed using two non-consecutive, 24-hour dietary recalls collected by trained nutritionists and entered into Nutrient Data System for Research (Minneapolis, MN, v. 2012) [37] Changes in physical activity will be measured over a 7-day period using Actigraph GT3X accelerometers (Actigraph, Pensacola, FL); raw accelerometer counts will be processed and analyzed using youth-specific cut-points [38].

Psychosocial measures will be evaluated to assess mediators of behavior change using validated questionnaires for youth including self-efficacy related to nutrition [39, 40] and physical activity [41], perceived competence in maintaining a healthy diet and exercising regularly [42], and perceived parental support [43]. Parents/caregivers will complete the Family Nutrition and Physical Activity Screening Tool [44] designed to assess environmental support for healthy eating and activity (e.g. provision of nutritious food and opportunities to be physically active), and will also self-report food parenting practices using the Comprehensive Feeding Practices Questionnaire [45]).

Physiological outcomes will be assessed through 12-hour fasting insulin, glucose, and lipids drawn at J2 Laboratories, Tucson, Arizona, by a trained phlebotomist and placed into serum separator vacuum tubes. Insulin resistance (IR) will be assessed using HOMA [46], shown to more sensitive for identifying youth with metabolic dysregulation than an impaired fasting glucose threshold [47]. Calculation of IR is based on modeling of fasting insulin and glucose concentrations using the formula: fasting insulin (μU/ml) × fasting glucose (mmol/L)/22.5 = HOMA. HOMA correlates closely to IR as measured by euglycemic clamp [48], and a HOMA insulin resistance value of 2.6 will be used as the upper limit of normal [49]. Blood pressure will be measured on the non-dominant arm using an automatic monitor (Omron HBP-1300, Hoofddorp, The Netherlands) and cuff-sizes appropriate for the mid-upper arm circumference. Systolic and diastolic blood pressure will be measured two times with 5 min intervals while the participant is seated and resting. The definition of hypertension will be adjusted for age per national guidelines [50]. Individuals with fasting glucose values >126 mg/dL and no previous history of T2D will be referred to their primary care provider. The study medical director will be immediately informed and arrange appropriate follow-up depending on the level of blood glucose elevation and symptoms. Maturity will be assessed using Tanner’s validated self-report questionnaire which presents illustrations of developmental stages shown to agree with pubertal staging by a physician [51, 52].

Feasibility data will include recruitment, enrollment, and retention rates, program attendance and engagement, and delivery costs. Program satisfaction will be self-reported by participants (parents and children) using brief surveys that inquire about relevance of content, promoters and barriers to attendance and engagement, and degree to which the family applied the intervention to lifestyle behavior changes. Adherence data will include attendance logs and time spent with mobile content (hybrid condition), and completion of self-monitoring and goal-setting activities (both conditions). Importantly, our mobile infrastructure [28] permits capture of engagement with mobile lessons including frequency and duration of participant log-ins, communication with lifestyle coaches, and content downloads/views, which will allow us to characterize engagement and associated it with adherence metrics.

### Data collection

Data collection and entry, and statistical analyses will be conducted by research staff who are blinded to treatment allocation. Participants will be assigned a study identification number upon enrollment. Study personnel involved in data collection will follow a strict written protocol that describes study measures for protecting confidentiality and privacy. All aspects of data collection and storage will be carefully monitored to ensure rapid detection of errors, inconsistencies or other problems. Data will be double entered with third party verification to insure data integrity, and kept in locked files and on a dedicated study computer and secure network. Access will be restricted to the principal investigator and designated staff. Files with information linking names and other personal data to participant identification numbers will be deleted at the end of the study, and written copies will be shredded.

### Statistical analysis

Outcome measures will be stratified by delivery format to assess whether the mobile technology-supported delivery of the intervention is as efficacious as the face-to-face format. Descriptive statistics for change in endpoints (mean, median, and standard deviation) between baseline and 12-week measurements will be computed separately for each program format (face-to-face or hybrid mobile technology-supported program). The statistical significance of the change at 12 weeks will be assessed using a paired t-test combining both formats. One-sided statistical tests will be used, as the interventions approaches are of interest only if they decrease the percentage overweight and lead to improvements in diabetes risk factors. Statistical significance is set at alpha = 0.05. Sample size constraints do not allow adequate statistical power to formally test for differences between the two formats. However, exploratory models using linear regression analysis will estimate the magnitude of the difference between the face-to-face and combined formats, adjusted for baseline levels and the potential correlation between the participants within a given YMCA (expected to be small). These parameter estimates will provide effect sizes to appropriately power a larger study comparing the two formats. Since this is a randomized comparison, confounding is not an issue as long as there is non-differential dropout between the two formats. Differential dropout between the two formats will be assessed using a Fisher’s Exact Test. Similar analyses will assess whether improvements are maintained after the intervention ends, by comparing the 24-week versus 12-week values. We acknowledge the potential for differential loss to follow-up and missing data. Baseline values of those who complete the study versus those who do not will be compared using two sample independent t tests and Fisher’s Exact Tests, as appropriate. Additional sensitivity analyses will use multiple imputation for missing values and an intent-to-treat analysis with baseline values carried forward.

### Anticipated results

We expect four to seven percent reduction in the percent of overweight youth after 12 weeks, similar to behavioral interventions in clinics [5, 53, 54]; we also anticipate significant reduction in waist circumference over 12 weeks. Expected behavioral outcomes in youth include reduced energy-dense food intake including less sugary-sweetened beverages, increased intake of vegetable and whole grain servings, increased daily moderate-to-vigorous physical activity, reduced sedentary media screen time, and improved sleep time (i.e. closer to suggested amounts of 9 h per night). Child self-efficacy, perceived competence, perceived parental support, and intrinsic motivation is also expected to improve, as is parental support of environmental changes in the home related to nutrition and physical activity and use of effective food parenting practices. Reductions in insulin resistance as measured by homeostasis model assessment (HOMA) and maintenance of weight loss/healthy weight trajectories after 24 weeks are anticipated [55]. Furthermore, evidence suggests mobile devices can support health behavior change [56, 57], and frequent interaction (made more feasible with mobile tools) between program and participant is a positive predictor of adherence [58]. Thus, we contend mobile devices provide similar opportunities for engagement and contact as face-to-face programs while reducing the burden associated with weekly attendance. We, therefore, expect similar results using a hybrid delivery (60 % face-to-face, 40 % mobile delivery) to be comparable to the traditional face-to-face instructor-led program.

### Process evaluation and monitoring

The intervention has an a priori focus on scalability, replication, dissemination, and sustainability. Process evaluation and monitoring plans were created using the RE-AIM framework, a tool to assess the public health impact of health promotion interventions as described by Glasgow et al. [59]. RE-AIM consists of five dimensions: reach, efficacy, adoption, implementation, and maintenance. These data will be collected using a combination of qualitative and quantitative measures and will involve our community partners (YMCA administrators and staff), the YMCA Lifestyle Coaches (delivering the intervention), and study participants. (Table 3) We will assess reach, impact, and engagement of participants and their families, and the feasibility of delivering the program using the YMCA infrastructure to inform future adoption, implementation and maintenance of the program. Efficacy will be measured both as the main study outcomes (anthropometric, behavioral and physiological, feasibility and acceptability, and attrition) and as the fidelity of the delivery of the intervention (e.g. training of and observations of curriculum delivery from Lifestyle Coaches). Quality assurance will occur with structured training and certification of YMCA lifestyle coaches, who will have continued access to the training team to discuss issues related to content or group moderation. The research team will review weekly session logs from YMCA instructors for potential departure from the EPIC Kids model, and conduct repeated observations of intervention sessions. Study staff trained in qualitative procedures will conduct interviews with YMCA staff and administrators to assess needs and resources for the program and for the YMCA at the program mid-point (November 2015). Engagement with administrators through interviews and surveys will inform activities related to the local adoption, implementation, and maintenance of EPIC Kids such as participant recruitment, branding and licensing, anticipated costs and resources associated with coach training, and program implementation and evaluation beyond the life of the grant.

**Table 3:** Process evaluation and monitoring plan

Conceptual category	Data collection instrument	Variables	When
Reach	Screening form	• Inquiries • Non-eligible • Recruitment/referral source	Screening
	Baseline questionnaire	• Participant demographics • New or past YMCA members • Motivation to join • Willingness to participate (distance to travel) • Competing programs	Enrollment
Efficacy	Lifestyle coach training survey & focus group	• Prior experience as a Lifestyle/DPP^a^ Coach • Comprehension of material • Knowledge and skills acquired • Self-efficacy/preparedness • Perceived barriers/ potential solutions • Motivation to lead • Acceptability (likes/ dislikes)	Pre-study training (1 time)
	Telephone debriefing with lifestyle coaches	• Ease of implementation • Barriers/potential solutions • Perceived participation • Time management	After first lesson (1 time)
	Lifestyle coach weekly survey	• Curriculum comprehension • Ease of implementation • Fidelity of lesson (delivered as written & on time) • Perceived self-efficacy • Motivation to lead again • Perceived participant participation • Perceived barriers/solutions • Acceptability (likes/dislikes)	Weekly after each lesson (11 times)
	Attendance record	• Adherence (in-person) • Mobile data analytics (adherence for mobile-device delivered group)	Weekly (12 times)
	Participant weekly questionnaire (Child, parent)	• Comprehension • New knowledge • Usefulness • Motivation • Likelihood to adopt (short & long-term) • Enjoyment • Acceptability (likes/dislikes) • Suggestions for improvement	Weekly after each lesson (11 times)
	UA staff observation rubric	• Study fidelity (delivered as written and on time) • Participant engagement (quantity & quality) • Instructor communication/organization • Instructor classroom management • Instructor support of student needs	4x each study site
	Lifestyle coaches final survey	• Perceived efficacy of intervention • Desire to continue program • Average preparation time • Time substitution (substituted activity if not involved in the intervention delivery) • Enjoyment & acceptability (likes/dislikes)	End of program (1 time)
	Participant final survey (Child and parent)	• Perceived effectiveness of instructor • Adoption of lifestyle changes due to program • Perceived long-term maintenance of changes • Likelihood to recommend program & perceived effectiveness of intervention for others • Use of YMCA for other purposes • Likelihood of future YMCA use • Cost of physical activity or food items due to intervention^b^ • Time substitution (activity substituted if they were not in the intervention)^b^	End of program (1 time)
	Study outcomes	• Anthropometric (BMI-percentile, waist circumference) • Behavioral (24-hr dietary recalls, accelerometry, psychosocial (e.g., self-efficacy) • Physiological (fasting insulin & glucose, blood lipids & pressure)	Pre-post study and follow-up 12 weeks later (3 times)
	N/A – program data	• Cost • Lifestyle Coach and Research Staff Training requirements (time, effort, effectiveness) • Recruitment source success rate • Quality assurance/control	Throughout intervention
Potential adoption, maintenance and implementation by YMCA^c^	YMCA administrator interview	• Likelihood of adoption • Perceived needs to implement and maintain • Suggestions for improvement	Perceived efficacy of intervention

^a^DPP = adult Diabetes Prevention Program

^b^Data collected from parents only

^c^Data from efficacy outcomes will be compiled post-study for a report to be presented to administrators prior to the interview

## Discussion

Obesity prevalence among U.S. youth remains high [1] heralding increases in T2D incidence and prevalence. The latest data suggest 18 % of 6–11 year-olds and 21 % of 12-19 year-olds have a BMI ≥ 95th percentile [1]. T2D, which has increased in prevalence along with pediatric obesity [13], accounts for approximately 45 % of new cases of T2D [60], an increase from 4 % in 2001. Behavioral interventions are the only real option for prevention of T2D in youth, given the cost, poor adherence and risks of pharmacological and surgical interventions [61]. Evidence-based, efficacious community interventions are critically needed.

EPIC Kids is an intervention that adapted the successful adult YMCA DPP to include evidence-based behavioral change strategies (e.g. goal setting, self-monitoring, promoting environmental changes [22]), while including fun, interactive, family-oriented, and physically active content led by YMCA instructors. The long-term goal of EPIC Kids is to create a scalable, replicable, and sustainable program with the YMCA that overcomes existing barriers to implementation and dissemination of evidence-based, research-proven diabetes prevention programs to youth and families, thereby improving population health.

## Abbreviations

BMI: Body mass index
RE-AIM: Reach, efficacy, adoption, implementation, and maintenance
YMCA: Young Men’s Christian Association
T2D: Type 2 diabetes mellitus
YDPP: YMCA diabetes prevention program
HOMA: Homeostasis model assessment
T1D: Type 1 diabetes mellitus
IR: Insulin resistance
IRB UA: Institutional Review Board of The University of Arizona
